# Native bacteria from a Mediterranean greenhouse associated to soil health and suppressiveness

**DOI:** 10.3389/fmicb.2025.1484219

**Published:** 2025-06-27

**Authors:** Jose Ignacio Marín-Guirao, Miguel de Cara-García

**Affiliations:** Andalusian Institute of Agricultural and Fisheries Research Training (IFAPA) La Mojonera, Almería, Spain

**Keywords:** agroecology, antagonism, biosolarisation, FAPROTAX, DNA metabarcoding, organic amendment, sustainability, *Trichoderma*

## Abstract

This study, conducted over two consecutive seasons in an organic-certified Mediterranean tomato greenhouse, aimed to assess the dynamics of soil bacterial composition at pre-planting phase following the incorporation of fresh sheep manure and subsequent solarisation (biosolarisation), as well as at the end of the crop cycle. Gene sequencing using 16S rRNA, and plate culture methods were applied. Additionally, dual culture tests were performed to evaluate the antagonistic activity of 95 soil-isolated bacteria against five soilborne pathogenic fungi and five beneficial fungi sourced from commercial products. Culturable and thermophilic bacterial populations shifted over time, but showed no clear trend. However, alpha diversity was lower at pre-planting phase and increased by the end of the cropping seasons. Significant shifts in beta diversity were also observed across sampling times. Firmicutes showed higher relative abundance at pre-planting phase, while Proteobacteria were consistently more abundant at the end of the cropping season. The genera *Bacillus* and *Thauera* were identified as biomarkers, with *Bacillus* associated with the pre-planting phase and *Thauera* with the end of the cropping seasons (LDA score > 4.5). Among the 52 ecological pathways detected via FAPROTAX database, nitrogen-related functions showed higher presence at the end of the cropping seasons. Isolates with antagonistic activity were detected at all sampling times. The 37.9% of the isolates showed antagonism, 31.6% against phytopathogenic fungi, 20.0% against beneficial fungi, and 12.6% against both. *Colletotrichum gloeosporioides* and *Botrytis cinerea* were the pathogens to which the highest number of isolates showed antagonism, while *Trichoderma asperellum* and *T. atroviride* were among the beneficial ones. Among the native antagonistic bacteria, *Streptomyces* spp. was the dominant genus followed by *Bacillus*. This information highlights how the functional diversity of native bacteria communities from biosolarised soils, may impact the performance of introduced biocontrol microorganisms, as well as the development of natural soil suppressiveness.

## 1 Introduction

The current trend on intensification in organic and conventional horticultural production systems, underscores the need for effective crop protection and production methods that ensure environmental sustainability (Lal, 2020). In this regard, disease-suppressive soils (DSS) represent a significant achievement toward sustainability. It refers to soils where disease progression remains minimal, even when soil-borne pathogens and susceptible host plants are present (Baker and Cook, 1974; Schneider, 1982), and is mainly influenced by the intrincate interactions among soil, plant, microbiome and environmental factors (Gómez Expósito et al., 2017; Jayaraman et al., 2021; Sagova-Mareckova et al., 2023). Although the abiotic factors of the soil are believed to play a role, soil biodiversity strongly influences the phenomena through diverse biological mechanisms which are summarized as (Hoitink and Boehm, 1999; Mazzola, 2002; Garbeva et al., 2006; Adesina et al., 2007; Jayaraman et al., 2021): (i) parasitism against pathogens, (ii) production of metabolites, toxins and antibiotics, (iii) competition for nutrients, resources and/or substrates, (iv) activation of disease-resistant genes in the host plant, and (v) improvement of plant nutrition and soil health. In this way, soil microbial community plays a crucial role in the DSS since it is involved in the above mentioned mechanisms (Alabouvette et al., 1993; Mazzola, 2002; Weller et al., 2002; Garbeva et al., 2004; Mazzola and Freilich, 2016; Siegel-Hertz et al., 2018).

Among the native soil microbiota, soil bacteria, including thermophilic bacteria, have been reported as main actors in the suppressiveness of soil-borne diseases in different agricultural soils (Mendes et al., 2011; Gómez Expósito et al., 2017; Schlatter et al., 2017). The composition of the bacterial community is one of the major factors in the mechanism of disease suppression (Hemkemeyer et al., 2024; Kang et al., 2024; Ni et al., 2022), and several bacterial genera including *Bacillus*, *Pseudomonas* and *Streptomyces*, among others, have been proposed as key players in disease suppressiveness of soils (Hoitink et al., 1997; Chng et al., 2015; Xiong et al., 2015; Eberlein et al., 2016). These microorganisms are actively involved in the transformation and decomposition of organic matter (Pankhurst and Lynch, 1995), and the incorporation of organic amendments (OA) can positively impact their communities, improving soil health and enhancing soil disease suppressiveness (Bonanomi et al., 2007, 2010, 2018; Postma and Schilder, 2015; Akanmu et al., 2021), as well as allowing to achieve greenhouse sustainability (Giagnocavo et al., 2022). In addition, OA incorporation impacts on physical and chemical factors that also has an influence on the mechanisms of soil suppressiveness (Amir and Alabouvette, 1993), as well as in greenhouse soil fertility (García-Raya et al., 2019; Marín-Guirao et al., 2019a, 2023a; Castillo-Díaz et al., 2021, 2022). In this regard, the incorporation of fresh organic matter followed by solarisation (i.e., biosolarisation) is an increasing practice in Mediterranean greenhouses, reaching 4.6% of producers in south-eastern Spain during campaign 2019/2020 (García et al., 2024). Biosolarisation is mainly practiced as a soil disinfection technique, but also to promote the decomposition of organic material, what helps to prevent potential phytotoxicity issues that can occur when seedlings are transplanted with fresh, insuficciently decomposed material.

Understanding the impact of soil biosolarisation on the native bacterial community is crucial, as it directly influences community structure and functionality. It is essential to appraises the extent of this impact, and to determine whether bacteria with suppressive properties against phytopathogenic fungi may emerge, following the implementation of such practices. Additionally, evaluating the persistence of these bacteria at the end of consecutive crop cycles is essential, as it is a highly relevant aspect in maintaining crop health (Mendes et al., 2011; Gómez Expósito et al., 2017). Furthermore, given the rising utilization of commercial products containing biological control organisms (BCO) and other beneficial microorganisms for crops, it is highly pertinent to study whether native soil bacteria might also exhibit a suppressive effect on these microorganisms. This currently limited understanding holds substantial significance and is crucial for the efficacy of such products when applied into the soil.

The present study was conducted for two consecutive years in an organic-certified greenhouse with tomato crops. The study aimed to assess the dynamics of soil bacterial composition at pre-planting phase following the incorporation of fresh sheep manure in summer and subsequent solarization (biosolarisation), as well at the finalization of the crop cycle, using 16S rRNA gene sequencing and plate culture methods on soil microbial composition. Additionally, dual culture tests were performed to evaluate the antagonistic activity of 95 soil-isolated bacteria collected at the start and end of the tomato crops against five soilborne pathogenic fungi and five beneficial fungi sourced from commercial products.

## 2 Materials and methods

### 2.1 Experimental greenhouse and soil management

The present study was conducted for two consecutive years (the 2019/2020 and 2020/2021 seasons) at the Andalusian Institute for Research and Training in Agriculture and Fisheries (IFAPA) in Almería, Spain (36°48′N, 2°41′W; altitude 142 m), the largest cropping region for Mediterranean greenhouses. The climate in the area is Mediterranean semi-arid, characterized by mild winters and hot summers with no rainfall. The experimental greenhouse, certified for organic production by the Andalusian Organic Farming Committee (C. A. A. E.) since 2006, covered 832 m^2^ and was representative of the “raspa y amagado” Mediterranean greenhouse. Two subsequent winter cycles of “Valenciano type” tomato plants (*Solanum lycopersicum* L.), grafted onto Armstrong® rootstock (Syngenta, Basel, Switzerland), were cultivated during 2019/20 and 2020/21 seasons, referred to as season 1 and season 2, respectively. Four-week-old tomato plants were planted on September 17th, 2019, and September 25th, 2020, in seasons 1 and 2, respectively. Prior to planting in both years, fresh sheep manure was uniformly incorporated into the greenhouse soil at a rate of 4 kg m^−2^ in July. Incorporation involved mixing the manure with the soil using a rotavator. Next, drip lines were deplyed and, with the aim of favoring manure’s decomposition, the soil was covered with transparent polyethylene film (30 μm TIF Desinfección DS^®^, Sotrafa, Almería, Spain) for a period of 2 months, after a single irrigation application to reach saturation at 15 cm depth. The maximum, minimum, and average temperatures at 20 cm soil depth during biosolarisation in 2020 were 44.8 ± 0.9, 31.8 ± 0.6, and 39.3 ± 0.3, respectively. Planting occurred 2 days after removing the film. Crop management and pest control practices were adhered to Regulation (EU) 2018/848 on organic production. At the start of the study, the soil consisted of 19% clay, 13% silt, and 68% sand. Soil pH was 8.57, electrical conductivity (EC) 6.19 dS m^−1^, organic matter content 1.20%, total carbonates (HCO_3_^−^) 14.33%, active limestone 5.67%, nitric-nitrogen (N) 157 mg L^−1^, total nitrogen 0.04%, phosphorus Olsen (P Olsen) 19 meq L^−1^, interchangeable calcium (Ca^+2^) 1,253 mg L^−1^, interchangeable sodium (Na^+^) 37 mg L^−1^, interchangeable magnesium (Mg^+2^) 261 mg L^−1^, interchangeable potassium (K^+^) 1,841 mg L^−1^, and C/N ratio 20. The soil had no history of soilborne pathogens.

### 2.2 Soil collection

Soil samples were taken at four different sampling times. During the 2-year study period, samples were collected 2 days before planting (i.e., Start Season 1, 15th September 2019, and Start Season 2, 23rd September 2020) and at the conclusion of the tomato crop season (i.e., End Season 1, 3rd April 2020, and End Season 2, 23rd April 2021). At Start Season 1, soil sampling was conducted in three designated areas of the greenhouse immediately preceding the planting of tomato seedlings. In subsequent sampling times, systematic soil samples were taken in 12 uniformly distributed plots within the greenhouse. The samples were taken with an auger at a depth of 0–30 cm. Three subsamples were randomly taken from cultivation lines and then mixed and homogenized to ensure representativeness in each sample. For each soil sample, one subsample was air-dried and sieved (<0.2 mm) for microbiological analysis through the plate culture method, and another subsample was frozen at −80°C for DNA extraction used in the assessment of bacterial microbiota through DNA metabarcoding.

### 2.3 Quantification and isolation of soil culturable bacteria and soil thermophilic bacteria

The preparation of soil samples included a drying, grinding, and sieving process, following the method described by Tello et al. (1991). Drying was conducted at room temperature (20–25°C) for a period of 7–10 days, until soil humidity was constant and homogenous. A porcelain mortar was used for grinding, and a 200 μm mesh-size sieve was used for sieving. Both the mortar and sieve were thoroughly washed and disinfected between samples by covering them with alcohol and lighting it. Then, the soil culturable bacteria and thermophilic bacteria were analyzed by means of the serial dilution method (Tello et al., 1991). The culture medium used for bacteria evaluation was nutrient agar (NA, Oxoid, Basingtoke, U. K.). In the case of thermophilic bacteria, soil samples were previously subjected to a heat treatment in an oven at 70°C for 1 h, using a semi-selective medium for actinobacteria (Crawford et al., 1993). This technique was applied to isolate the live thermophilic culturable fraction and to allow the study of its antagonistic activity (see Section 2.5.). In both cases, five subreplicates (9 cm-diameter Petri dishes) of each soil sample were analised at 10^−5^ and 10^−6^ dilutions. The Petri dishes were incubated at 25°C for 7 days. Subsequently, total colony forming units (CFU) of bacteria and thermophilic bacteria were quantified, eventually expressing the results as CFU/g dry soil.

### 2.4 Assessment of bacterial microbiota through DNA metabarcoding

#### 2.4.1 Soil DNA extraction, amplicon high-throughput sequencing, and processing of sequencing data

DNA from soil samples was isolated using the DNeasy PowerSoil Pro DNA isolation kit (Qiagen, Hilden, Germany), strictly following the manufacturer’s instructions. An extraction blank for cross-contamination was included. DNA was quantified using the Qubit High Sensitivity dsDNA Assay (Thermo Fisher Scientific, Waltham, MA, United States). For bacterial library preparation, a fragment of the 16S genomic region (of about 460 bp) was amplified using the primers Bakt_341F (5′ CCT ACG GGN GGC WGC AG 3′) and Bakt_805R (5′ GAC TAC HVG GGT ATC TAA TCC 3′) (Herlemann et al., 2011). Illumina sequencing primers were attached to these primers at their 5′ ends. Polymerase chain reactions (PCRs) were carried out in a final volume of 12.5 μL, containing 1.25 μL of template DNA, 0.5 μM of the primers, 3.25 μL of Supreme NZYTaq 2x Green Master Mix (NZYTech, Lisbon, Portugal), and ultrapure water up to 12.5 μL. The reaction mixture was incubated as follows: an initial denaturation step at 95°C for 5 min, followed by 25 cycles of 95°C for 30 s, 50°C for 45 s, 72°C for 45 s, and a final extension step at 72°C for 7 min. A negative control that contained no DNA (BPCR) was included in every PCR round to check for contamination during library preparation. The libraries were run on a 2% agarose gel stained with GreenSafe (NZYTech) and imaged under UV light to verify the library size. Libraries were purified using the Mag-Bind RXNPure Plus magnetic beads (Omega Biotek, Beijing, China), following the instructions provided by the manufacturer. Then, libraries were pooled in equimolar amounts according to the quantification data provided by the Qubit dsDNA HS Assay (Thermo Fisher Scientific). This pool also contained a testimonial amount (1 μL) of the PCR blank and DNA extraction blank (Bex). The pool was sequenced in the MiSeq PE300 run (Illumina). DNA metabarcoding analyses were carried out by AllGenetics & Biology S. L. (La Coruña, Spain; www.allgenetics.eu). Illumina paired-end raw forward (R1) and reverse (R2) FASTQ reads were stored in separate files. The quality of the FASTQ files was checked with the software (v0.11.9) FastQC (Andrews, 2010) and the output was summarized using MultiQC (Ewels et al., 2016). The obtained amplicon reads were processed using QIIME 2 (release 2022.2) (Bolyen et al., 2019). Specifically, the tool DADA2 (Callahan et al., 2016) (implemented in QIIME 2) was used to remove the PCR primers, filter low-quality reads, denoise, merge the forward and reverse reads, remove the chimeric reads, and cluster the resulting sequences into amplicon sequence variants (ASVs). The resulting output of the DADA2 pipeline was a table containing the number of occurrences of every observed ASV in each sample. The taxonomic assignment of ASVs was conducted using a pre-trained classifier of the SILVA reference database (Quast et al., 2013) (updated in August 2020). Specifically, the feature-classifier classify-sklearn approach implemented in QIIME 2 (Bokulich et al., 2018) was employed. The following ASVs were excluded: singletons (i.e., ASVs containing only one member sequence in the whole dataset), ASVs occurring at a frequency below 0.01%, non-bacterial ASVs such as eukaryotic sequences of chloroplast (Moore et al., 2019) and mitochondrial (Emelyanov, 2001) origin, unidentified sequences, and sequences assigned only at the kingdom level. The final filtered ASV table was converted into a Biological Observation Matrix (biom) file that was directly imported into R 3.6.1 (R-Team-Core, 2019) using the package phyloseq 1.24.2 (McMurdie and Holmes, 2013). The final filtered ASV and the biom file were used for further analysis and plotting.

#### 2.4.2 Bacterial community description and data processing

Four indexes of biodiversity were selected to study the alpha diversity of the bacterial community of each sample: Margalef genus richness (d) (Margalef, 1958); the Shannon–Wiener diversity index (H′) log basis (Shannon and Weaver, 1949); Pielou’s evenness (J) (Pielou, 1969) of the distribution of individuals among genera; and Simpson’s dominance (D) (Simpson, 1949). All calculations were carried out with the software PRIMER version 6.0 (Primer-E Ltd., Plymouth, U. K.) for Windows (Clarke and Gorley, 2006). Regarding beta diversity, bacterial community data at the operational taxonomic unit (OTU) level through DNA metabarcoding evaluation method, were compared among sampling times using permutational analysis of variance (PERMANOVA). Also, pairwise PERMANOVA comparisons were performed. These tests calculate Pseudo-F statistics to obtain *p*-values (Anderson, 2001). All PERMANOVA tests used 9,999 permutations from unrestricted permutations of raw data. Previously, data were square root transformed. The calculation of similarity matrices in the PERMANOVA analysis was based on Bray–Curtis distance. In addition, principal coordinate analysis (PCoA) was used to visualize the potential differences in the soil bacterial community structure among sampling times or within sampling times. PERMANOVA and PCoA analyses were performed using the multivariate statistical software package PRIMER-6 with the PERMANOVA+ extension (Primer-E Ltd., Plymouth, UK) for Windows. The linear discriminant analysis effect size (LEfSe) algorithm (Segata et al., 2011) was generated from Python (version 3.12) and used to identify and compare unique bacterial taxa significantly related to any of the four sampling times (Start Season 1, End Season 1, Start Season 2, End Season 2). In addition, data from Start Season 1 and Start Season 2, as well as those from End Season 1 and End Season 2, were pooled in order to identify and compare unique bacterial taxa significantly related to Start Season and End Season, respectively. The threshold for the logarithmic linear discriminant analysis (LDA) score was established at 4.5 and the Wilcoxon *p*-value was set at 0.05.

Bacterial functional potential was investigated using Functional Annotation of Prokaryotic Taxa (FAPROTAX) database (Louca et al., 2016). FAPROTAX is a database that categorizes bacterial taxa into recognized metabolic or ecologically significant groups (considered as functional groups), utilizing current literature on cultured strains as a reference. Following the procedure of Vido et al. (2024), previously bacterial ASVs was filtered to reduce noise (i.e., removal of those with <10 counts). In addition, filtering was completed by removing functional groups with reads abundances ≤100. The abundances of each taxa assigned to an functional group were pooled to form a total relative abundance count for each group, and the abundances of unassigned taxa were excluded. Then, the diversity and composition of ecological groups within each sampling time were compared and contrasted using the same method outlined for taxonomy beta diversity. Thus, PERMANOVA, pairwise PERMANOVA and PCoA were conducted on the top 15 ecological groups to focus on the most relevant ecological pathways. In addition, LEfSe was performed to detect differentially abundant ecological pathways across groups of soil samples depending on sampling time (LDA score established at 3).

### 2.5 *In vitro* antagonism toward phytopathogenic and beneficial fungi

#### 2.5.1 Strains isolation and target fungi

The isolates used in the antagonism assays were obtained from the thermophilic bacteria analyses described in section 2.3. Following the evaluation specified in that section, colonies from soil samples taken at different sampling times were randomly selected and isolated on Petri dishes with 15 mL of NA medium. Eleven isolates from Start Season 1, 51 from End Season 1, and 33 from Start Season 2 were selected. Thus, the antagonistic activity of 95 soil-isolated bacteria against nine fungi and one oomycete (*Phytophthora capsici*), referred to as fungi throughout the rest of the document, was studied. Specifically, the evaluations were conducted against five soilborne pathogenic fungi, as well as five beneficial fungi sourced from commercial products (Table 1).

**Table 1:** Phytopathogenic and beneficial fungi used in dual culture tests and origin of the strains.

Target fungi used in the *in vitro* antagonism assays	Code	Origin
Pathogenic fungi		
*Colletotrichum gloeosporioides*	Cg	Marín-Guirao et al. (2023b)
*Fusarium proliferatum*	Fp	Brizuela et al. (2020)
*Fusarium oxysporum* f. sp. *radicis-cucumerinum*	Forc	Águila-Carricondo et al. (2024)
*Botrytis cinerea*	Bc	Marín-Guirao et al. (2023b)
*Phytophthora capsici*	Pc	Águila-Carricondo et al. (2024)
Beneficial fungi		
*Trichoderma harzianum* T-22	Th	Koppert B. V.
*Trichoderma longibrachiatum*	Tl	BIOMIP S. L.
*Trichoderma asperellum*	Ta1	Certis Europe B. V.
*Trichoderma atroviride*	Ta2	Certis Europe B. V.
*Paecilomyces lilacinus*	Paec	Certis Europe B. V.

#### 2.5.2 Dual culture protocol

Dual culture tests on Potato Glucose Agar (PGA, Oxoid, Basingtoke, U. K.) were performed to evaluate the antagonistic activity of soil-isolated bacteria against the ten target fungi (Table 1). Plates (9 cm diameter), which contained 15 mL of PGA, were inoculated with strain culture by streak and incubated at 25 ± 1°C in the dark for 8 days. Later, a 5-mm-diameter disk of the target fungi was placed at an equidistant distance between the bacteria and the edge of the plate. The disk was obtained from the edge of the colony where the growth is most active. The initial distance between the bacteria and the fungus was 3.25 cm, and the bacteria were streaked up to 2 cm from the edge of the plate. The plates containing the bacteria and the fungus in confrontation were sealed and incubated at 25 ± 1°C in darkness until the fungus reached the edge of the plate opposite to the bacteria. At this point, measurements were performed. The distance between the edge of the bacterial growth and the edge of the fungal colony (inhibition distance) was measured. The approximate incubation times varied depending on the target fungus: 3–4 days for *Botrytis cinerea*, *Trichoderma longibrachiatum*, *Trichoderma harzianum* T-22, and *Trichoderma atroviride*, 7–8 days for *Fusarium oxysporum* f. sp. *radicis-cucumerinum* (FORC), *Fusarium proliferatum*, *Colletotrichum gloeosporioides*, and *Trichoderma asperellum*, and 7–10 days for *Phytophthora capsici*, while *Paecilomyces lilacinus* exhibited the slowest growth, taking 20–30 days until measurement. Antagonistic activity was considered when the inhibition distance was equal to or greater than 4 mm to any of the target fungus, and additionally, the growth of the target fungi did not continue after the 5-day observation period. Five replicates were prepared for each combination bacteria-fungi.

#### 2.5.3 Identification of soil-isolated bacteria with suppressive properties

The isolates that showed antagonistic activity were analyzed by means of PCR sequencing of amplicons of the 16S rRNA gene using the primers 27F-1492R (Condori et al., 2019) and subsequent database searches using BLASTN software, based on the consensus sequences created by aligning the forward and reverse sequences of the target isolates. The PCR conditions were 5 min at 94°C, 35 cycles of 1 min at 94°C, 1 min at 55°C, 2 min at 72°C, and a final elongation of 7 min at 72°C. Purified PCR products were sequenced using the Sanger sequencing method (Instituto de Biología Molecular y Celular de Plantas, Valencia, Spain) and edited via base calling on the BioEdit program (Hall, 1999).

### 2.6 Statistical analysis

Data on the total culturable bacteria and thermophilic culturable bacteria populations, sequencing depth for the 16S library, the relative abundance of dominant bacterial phyla and genera, and the indices of biodiversity were analyzed via an analysis of variance (one-way ANOVA) to compare differences among sampling times. Previously, normality and homoscedasticity were tested using the Shapiro–Wilk and Levene tests, respectively. The Kruskal–Wallis one-way non-parametric test (*p* = 0.05) was performed when normality or homoscedasticity of data was not evident (*p* < 0.05 Shapiro–Wilk or Levene tests, respectively). Arcsine square root transformation was applied to percentages before analyses. Duncan’s *post hoc* test was then applied to perform a pairwise comparison between sampling times at a 95% confidence level. These statistical analyses were carried out using the statistical software package Statgraphics Centurion XIX (Statgraphics Technologies, Inc., The Plains, VA, United States) for Windows (Microsoft Corporation, WA, United States).

## 3 Results

### 3.1 Taxonomic compositions of the bacterial communities

**Figure 1:**
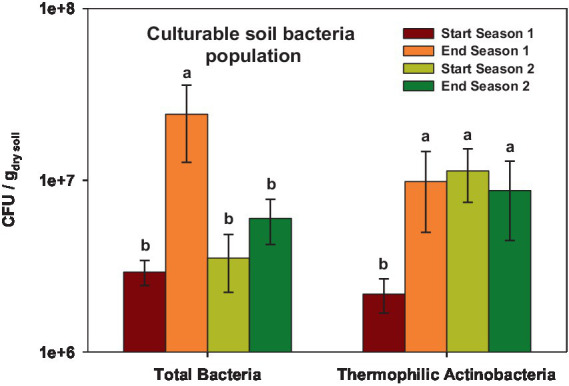
Total Colony Forming Units (CFU) of soil culturable bacteria and thermophilic bacteria at the start and at the end of season 1 (2019/20) and season 2 (2020/2021). In each case, different letters denote significant differences between sampling times at *p* ≤ 0.05 (Duncan’s test).

The total culturable bacteria population exhibited values ranging from 2,920,000 to 24,200,000 CFU/g dry soil depending on the sampling time (Figure 1). The highest values were observed at the end of Season 1, which showed significant differences (*p* < 0.001) compared to the other sampling times. For thermophilic culturable bacteria, the population ranged from 2,170,000 to 11,300,000 CFU/g dry soil. In this case, the population was lower at the beginning of Season 1 (*p* < 0.05) compared to the other sampling times (Figure 1).

**Figure 2:**
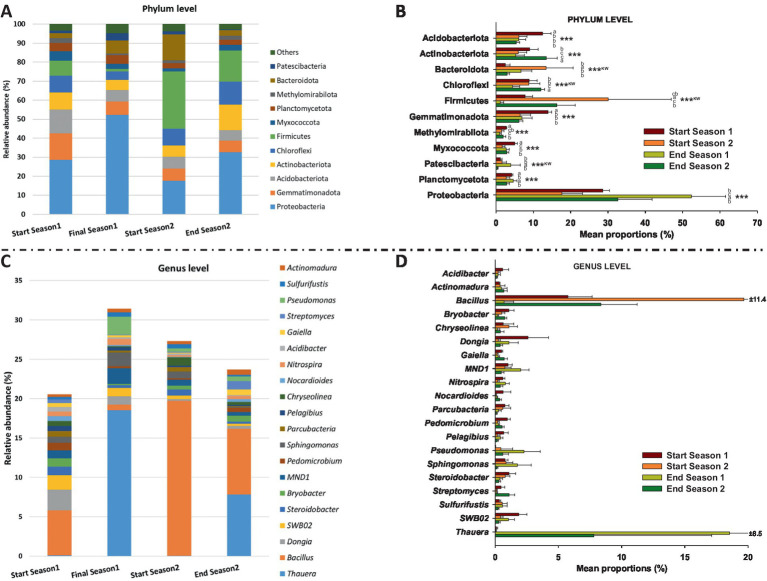
Bacterial community data found in the soil by 16S RNA sequencing. Relative abundance (%) of dominant bacterial phyla **(A,B)** and genera **(C,D)** divided into the start and end of the 2 years (seasons). “***” indicates significance at *p* ≤ 0.001 (Duncan’s test). “^KW^” *p*-value through the Kruskal–Wallis non-parametric test. Note: All genera in **(D)**, except *Acidibacter* sp., showed significance at *p* ≤ 0.01 through the Kruskal–Wallis non-parametric test.

Figure 2 illustrates the relative abundance of different microbial phyla (Figures 2A,B) and genera (Figures 2C,D) at the start and at the end of the two seasons. Regarding phyla, Proteobacteria dominated in both start and end seasons, with a significant increase (*p* < 0.001) from 28.6% at the start to 52.3% at the end in season 1, and from 17.6% at the start to 32.6% at the end in season 2. Firmicutes showed an increase from 7.8% in start season 1 to 30.1% in start season 2, followed by a decrease to 16.3% in end season 2 (*p* < 0.001). Actinobacteriota also showed fluctuations (*p* < 0.001), peaking at 13.5% in end season 2. Acidobacteriota, Gemmatimonadota, Methylomirabilota, and Myxococcota showed the highest relative abundance in start season 1 (*p* < 0.001). Other phyla, such as Bacteroidota, Planctomycetota, Patescibacteria, Chlorofexi displayed varying patterns but maintained presence throughout the two seasons.

Similarly, the relative abundance of bacterial genera varied across seasons (Figure 2D). *Thauera* experienced increase in both seasons, rising from 0.09 to 18.55% in Season 1 and from 0.03 to 7.80% in Season 2. In contrast, *Bacillus* showed decrease, dropping from 5.74 to 0.69% in Season 1 and from 19.68 to 8.36% in Season 2. Other notable trends include MND1, which increased from 1.00 to 2.00% in Season 1. Some genera, like *Pseudomonas*, exhibited notable fluctuations, with a peak of 2.26% in End Season 1. Others, such as *Streptomyces, Dongia* and SWB02 showed sporadic occurrences throughout the seasons, with values ranging from 0.09 to 1.09%. All genera, except *Acidibacter*, showed significant differences among sampling times (*p* ≤ 0.01) with no common pattern among them.

### 3.2 Alpha diversity of soil bacterial community

**Figure 3:**
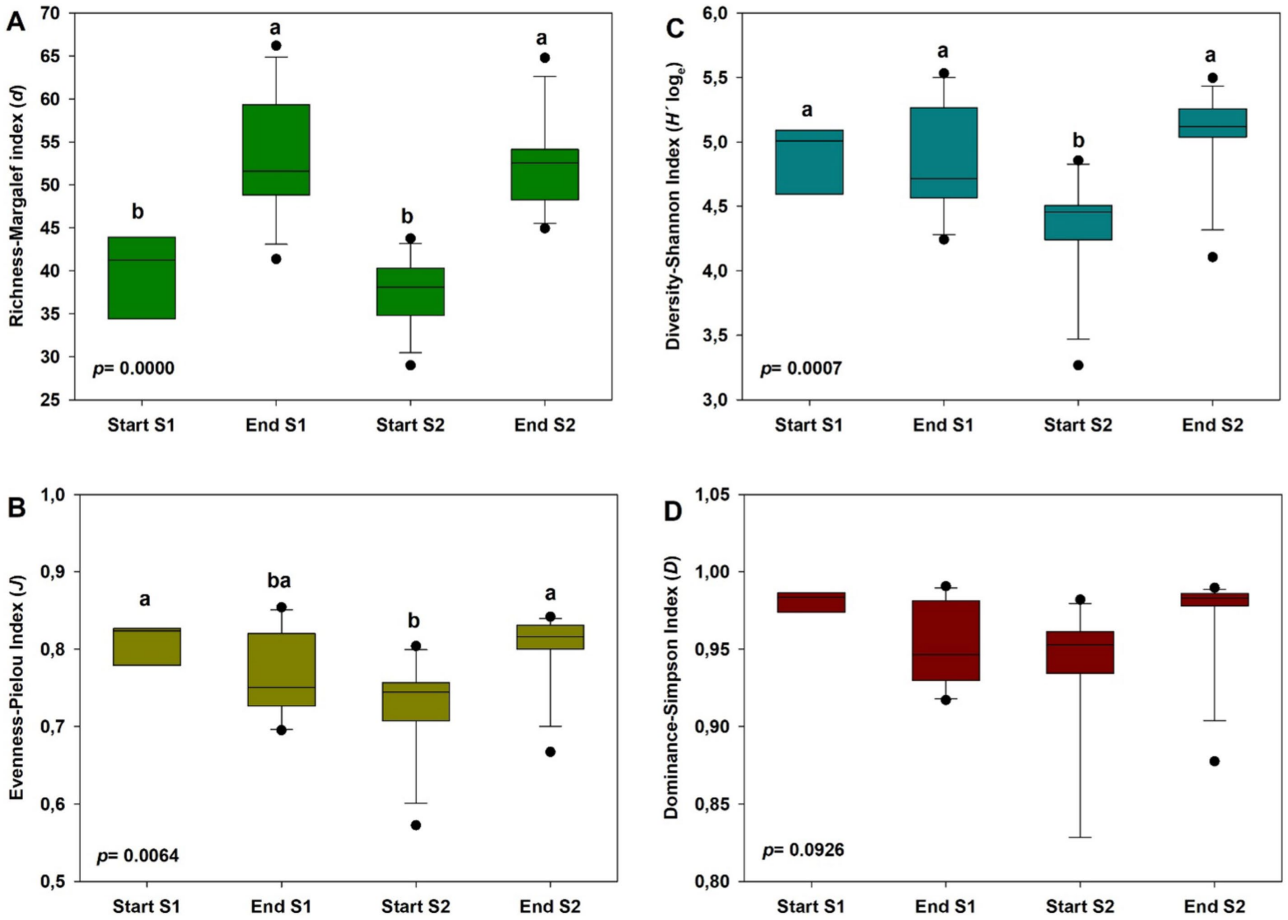
Alpha diversity indices of soil bacterial community at the start and at the end of season 1 (S1; 2019/20) and season 2 (S2; 2020/2021): Margalef richness index **(A)**, Shannon–Wiener diversity index **(B)**, Pielou’s evenness index **(C)** and Simpson’s dominance index. In each case, different letters denote significant differences between sampling times at *p* ≤ 0.01 (Duncan’s test).

Generally, the alpha diversity indices showed significant differences among different sampling times (*p* < 0.01), except for the Dominance-Simpson index (Figures 3A–D). Thus, species richness (Margalef index) was higher at the end of the crop in both years compared to values at the start of the crops (*p* = 0.0000), with average values ranging from 53.30 ± 7.01 to 37.59 ± 3.99 (Figure 3A). The Evenness-Pielou Index did not offer this trend, showing higher values at the start and end of the study compared to values at the start of the second year (*p* = 0.0064), although these differences were not detected at the end of the crop in year 1. In the case of the Diversity-Shannon index, values were lower at the start of the crop in year 2 compared to the other time points considered (*p* = 0.0007), with average values ranging from 4.35 ± 0.41 to 5.07 ± 0.35.

### 3.3 Beta diversity of soil bacterial community. Comparison of soil bacterial community structure among sampling times

The global PERMANOVA test indicated a significant effect (Pseudo-*F* = 9.6531, *p* < 0.0001) of the sampling time on bacterial communities’ beta diversity. In addition, pairwise tests revealed significant differences between bacterial communities across different seasons (Supplementary Table S1). Notably, comparisons between the start and end of each year exhibit considerable dissimilarities (*p* < 0.003). However, comparisons between the different sampling times highlight significant variations in bacterial composition in all cases (*p* < 0.0001).

**Figure 4:**
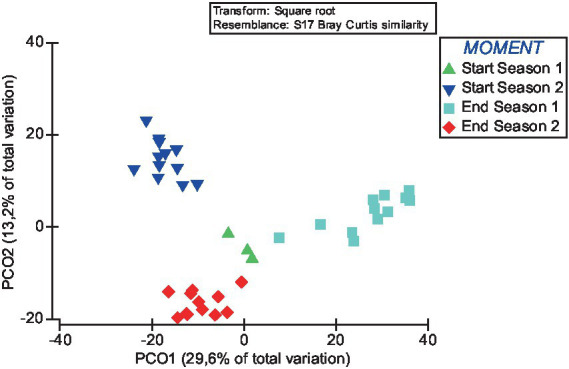
Principal coordinate analysis (PCoA) of the influence of sampling time on the beta diversity of soil bacterial communities. Over the 2 years of the study, soil samples were taken at the start of the crop (Start Season: after incorporating fresh sheep manure in summer followed by solarisation) and at the end (End Season).

Principal coordinates analysis (PCoA) further supports the statistical findings of the PERMANOVA analysis. The PCoA results show that the PCoA1 and PCoA2 explained 29.6 and 13.2% of the difference in the compositions of the bacterial communities among samples (Figure 4). The results of PCoA showed that the bacterial communities in the samples of the four different sampling time had a discrete distribution, with relatively large distances among the samples of each moment and a clear clustering of samples from each time.

### 3.4 Compositional differences and biomarkers of the bacterial communities

**Figure 5:**
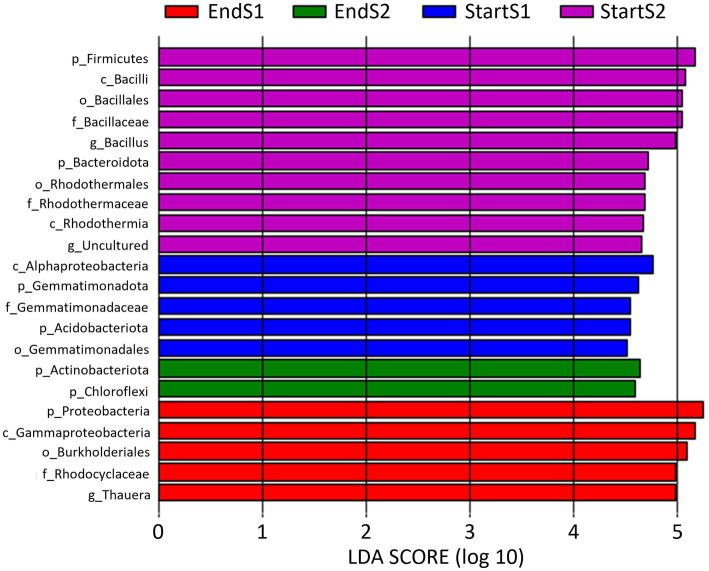
Linear discriminant analysis (LDA) to detect differentially abundant taxa across groups of soil samples depending on sampling time: start and end of season 1 (S1; 2019/20) and season 2 (S2; 2020/2021). Only taxa with an LDA score >4.5 (*p* < 0.05) are shown.

LEfSe analysis identified biomarkers that caused significant differences among the four sampling times (Figure 5). A total of 5, 10, 5, and 2 biomarkers (LDA score > 4.5) were identified in the soil bacterial community analyzed by means of DNA metabarcoding at the Start Season 1, Start Season 2, End Season 1 and End Season 2, respectively.

**Figure 6:**
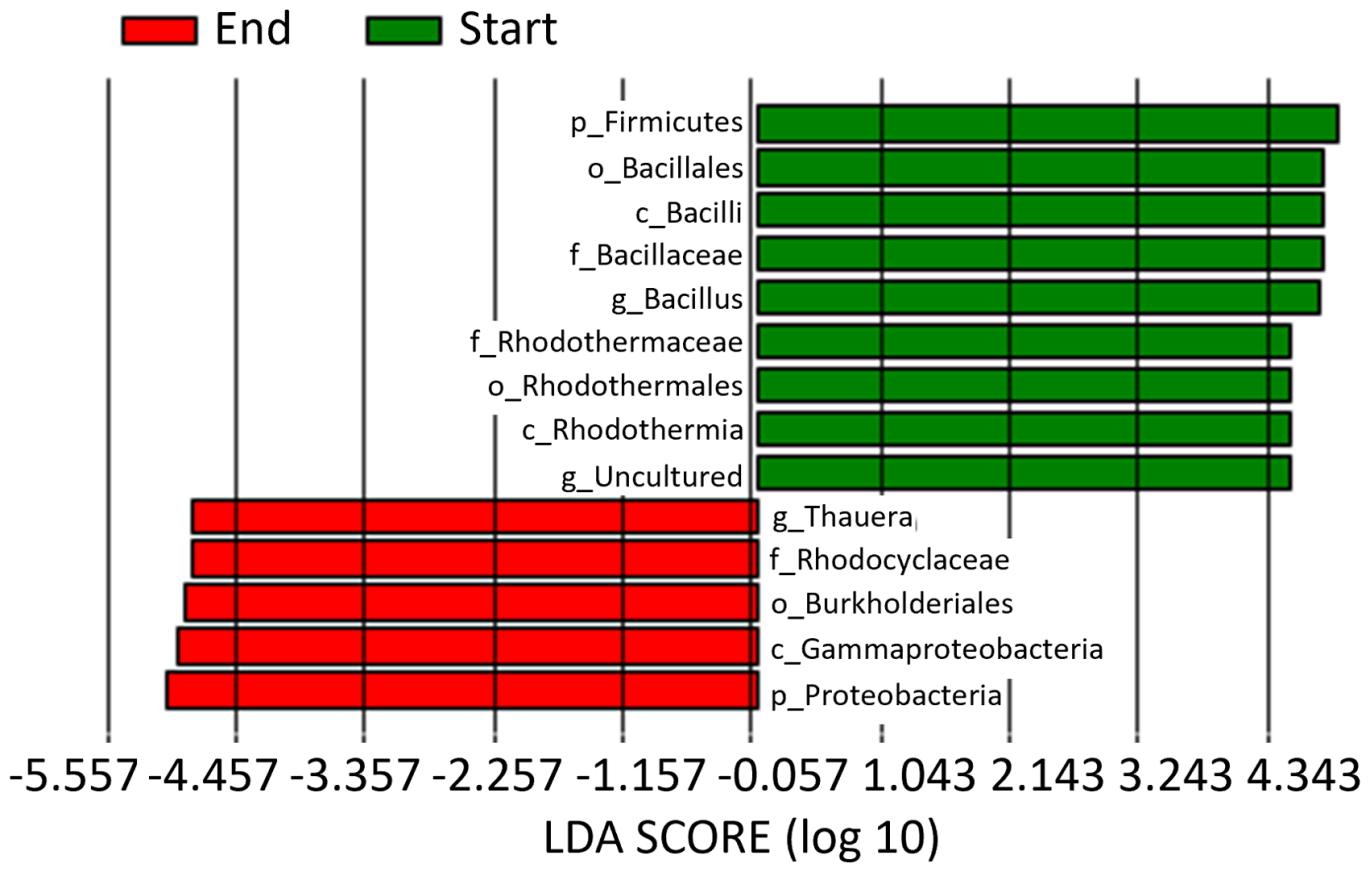
Linear discriminant analysis (LDA) to detect differentially abundant taxa across groups; only taxa with an LDA score >4.5 (*p* < 0.05) are shown. Data from the 2 years of study are pooled based on whether they come from soil samples taken at the start (Start) or at the end (End) of the seasons.

When data from the 2 years of study are pooled based on whether they come from soil samples taken at the start or at the end of the seasons, a total of 9 and 5 biomarkers were identified at the beginning and end of the crop, respectively (Figure 6). Thus, the phylum Firmicutes, class Bacilli, order Bacillales, family Bacillaceae, and genus *Bacillus*, along with the class Rhodothermia, order Rhodothermales, family Rhodothermaceae, and an uncultured genus, were the biomarkers identified at the start of the crop. Whereas at the end of the crop, they were the phylum Proteobacteria, class Gammaproteobacteria, order Burkholderiales, family Rhodocyclaceae, and genus *Thauera*.

### 3.5 Soil bacterial function prediction

The abundances of different bacterial functional pathways were predicted using the FAPROTAX database.

#### 3.5.1 Functional annotation overview based on 16S rRNA data

From a total sequencing reads average per sample of 56,976, an average of 29,763 reads per sample received functional assignment to any of the 52 functional groups detected via FAPROTAX database. This represents 45 ± 23.4% of the total sequencing reads. Considering the percentage of readings assigned to functionality at the different sampling times, the highest value was observed in the End Season 1 (68.9 ± 15.7%), which showed differences compared to the other sampling moments (*p* ≤ 0.001; Duncan’s test), including End Season 2, which presented an average value of 50.4 ± 14.4%. It is noteworthy that the lowest values were detected at the Start Season in both years of study, with values of 22.0 ± 4.2% and 21.4 ± 5.8% in Season 1 and Season 2, respectively.

#### 3.5.2 Dominant functional groups and their temporal distribution

**Figure 7:**
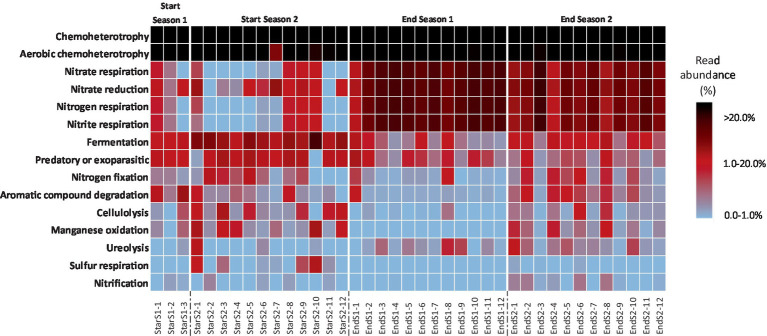
Relative abundance heat map showing the relative abundance of 15 bacterial functional groups within the bacterial communities found in the soil at each sampling time. Functional groups were normalized within each sample via total sum scaling resulting in a color scale denoting the proportion within each sample.

**Figure 8:**
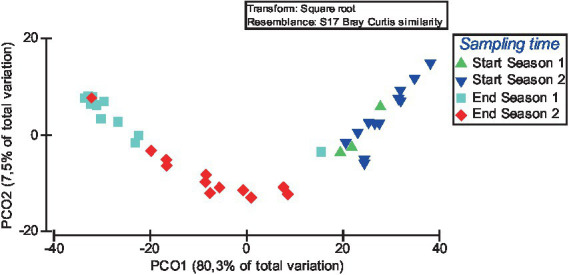
Principal coordinate analysis (PCoA) of the influence of sampling time on the composition and diversity of the top 15 bacterial functional groups. Over the 2 years of the study, soil samples were taken at the start of the crop (Start Season: after incorporating fresh sheep manure in summer followed by solarisation) and at the end (End Season).

Based on the FAPROTAX database, the top 15 functional groups which represented an average of 97.1 ± 2.2%, 87.2 ± 15.5%, 95.2 ± 2.7% and 92.7 ± 6.3% of the total sequencing reads assigned to functionality of Start Season 1, Start Season 2, End Season 1 and End Season 2 soil samples, respectively, were obtained. The relative proportion (i.e., percentage) of these top 15 functional groups across all sampling times is showed as a heatmap (Figure 7). In all sampling times, chemoheterotrophy emerges as the primary ecological pathway, representing more than 20% of the sequences in all cases, and surpassing 50% in some of them. This is followed by aerobic chemoheterotrophy, which similarly exhibits values above or close to 20%. It is remarkable that no entry was found for group “plant pathogen.” Figure 7 represents similarities in the relative abundance of the 15 bacterial functional groups within the soil bacterial communities at each sampling time, particularly between Start Season 1 and Start Season 2, as well as between End Season 1 and End Season 2. Nitrogen respiration, nitrate respiration, nitrite respiration, and nitrate reduction are observed with higher relative abundance in the samples from End Season 1 and End Season 2, whereas Fermentation emerges as the ecological pathway that appears to be more relevant in the samples from Start Season 1 and Start Season 2. In this regard, When the global PERMANOVA test was conducted, a significant main effect (Pseudo-*F* = 31.467, *p* < 0.0001) of sampling time on the composition of functional groups was detected (Supplementary Table S2). However, the only case where pairwise PERMANOVA tests did not find significant differences was between the samples from Start Season 1 and Start Season 2. Principal Coordinates Analysis supported the statistical findings of the PERMANOVA analysis. In this case, the PCoA results indicated that PCoA1 and PCoA2 explained 80.3 and 7.5% of the differences in compositions and diversity of the bacterial functional groups (Figure 8). The results of PCoA showed that the functional bacterial groups in the samples of the four different sampling time had a discrete distribution, with relatively large distances among the samples of each moment and a clear clustering of samples from each sampling time, except for the samples from Start Season 1 and Start Season 2, which exhibited mixing. Although pairwise PERMANOVA tests found differences between End Season 1 and End Season 2, the PCoA results showed all samples from End Season of both years separated from those of Start Season of both years along PCoA1.

#### 3.5.3 Differential functional pathways identified by LEfSe

**Figure 9:**
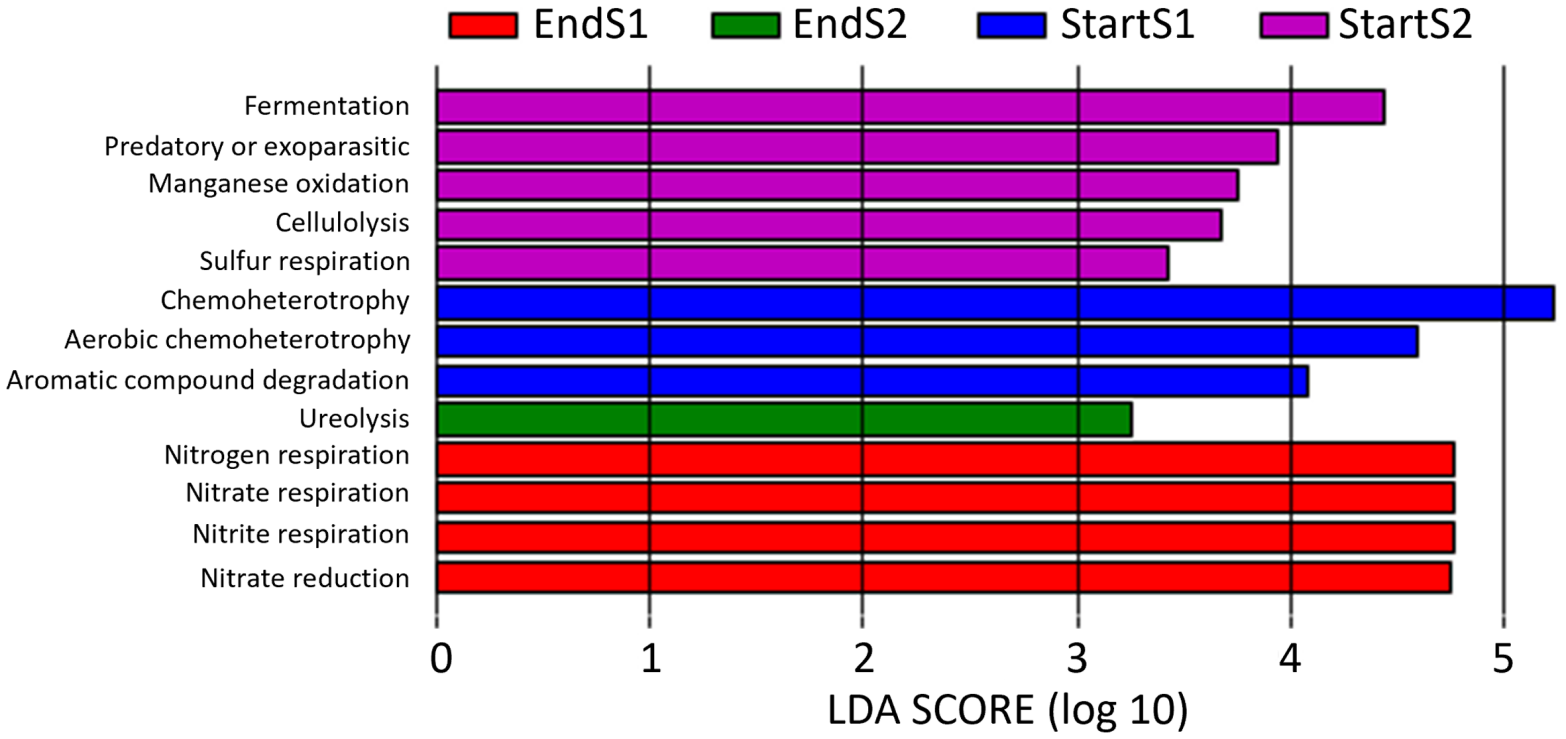
Linear discriminant analysis (LDA) to detect differentially abundant ecological pathways across groups of soil samples depending on sampling time: start and end of season 1 (S1; 2019/20) and season 2 (S2; 2020/2021). Only ecological pathways with an LDA score >3 (*p* < 0.05) are shown.

LEfSe analysis identified a total of 13 functional pathways that caused significant differences (LDA score > 3) among the four sampling times (Figure 9): Aromatic compound degradation, Chemoheterotrophy and aerobic chemoheterotrophy at Start Season 1; fermentation, predatory or exoparasitic, manganese oxidation, cellulolysis y Sulfur respiration at Start Season 2, nitrogen, nitrate and nitrite respiration and nitrate reduction at End Season 1, and ureolysis at End Season 2.

**Figure 10:**
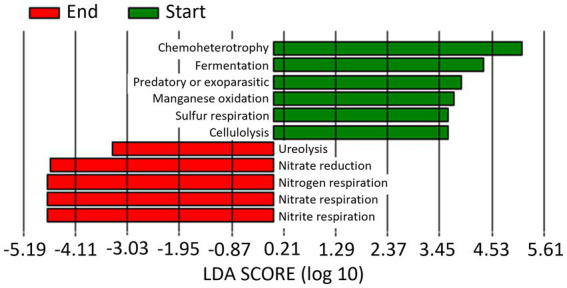
Linear discriminant analysis (LDA) to detect differentially abundant ecological pathways across groups; only ecological pathways with an LDA score >3 (*p* < 0.05) are shown. Data from the 2 years of study are pooled based on whether they come from soil samples taken at the start (Start) or at the end (End) of the seasons.

When data from the 2 years of study are pooled based on whether they come from soil samples taken at the start or at the end of the seasons, a total of 6 and 5 markers were identified at the start and end of the season, respectively (Figure 10). Thus, Chemoheterotrophy, fermentation, predatory or exoparasitic, manganese oxidation, cellulolysis and sulfur respiration were the biomarkers identified at Start Season, while nitrogen, nitrate and nitrite respiration, nitrate reduction and ureolysis at End Season.

### 3.6 Antagonistic bacteria against the *in vitro* growth of phytopathogenic and beneficial fungi

**Figure 11:**
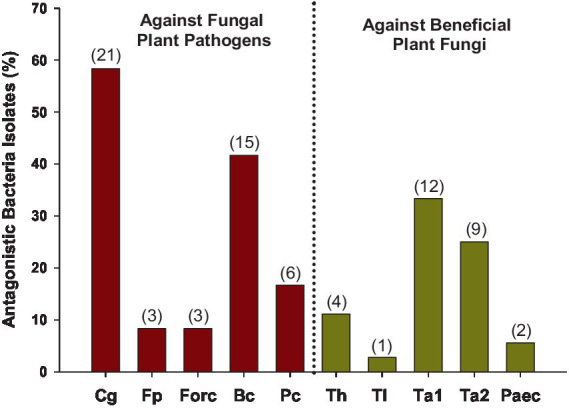
Soil-isolated bacteria with antagonism against the tested fungi. In total, 95 isolates were evaluated. Percentages expressed over the total bacteria that exhibited antagonism, which were 36. In brackets, the number of isolates with antagonism against each fungus. Only those with the inhibition distance equal to or greater than 4 mm to the target fungus were considered. Cg: *Colletotrichum gloeosporioides*; Fp: *Fusarium proliferatum*; Forc: *Fusarium oxysporum* f. sp. *radicis-cucumerinum*; Bc: *Botrytis cinerea*; Pc: *Phytophthora capsici*; Th: *Trichoderma harzianum* T-22; Tl: *Trichoderma longibrachiatum*; Ta1: *Trichoderma asperellum*; Ta2: *Trichoderma atroviride*; Paec: *Paecilomyces lilacinus.*

In total, out of the 95 isolates tested, 36 of them (37.9%) showed antagonism, 30 (31.6%) against phytopathogenic fungi, 19 (20.0%) against beneficial fungi, and 12 (12.6%) against both (Table 2; Figure 11). *Colletotrichum gloeosporioides* and *Botrytis cinerea* were the pathogenic fungi against which the highest number of isolates showed antagonism, with 58.3 and 41.7% of the isolates showing antagonism, respectively. Meanwhile, *Trichoderma asperellum* and *T. atroviride* were prominent among the beneficial fungi, with 33.3 and 25.0% of the isolates, respectively. Only 8.3% exhibited antagonism against *F. proliferatum* and Forc, while *T. longibrachiatum* and *Paecilomyces lilacinus* were the fungi with the least antagonistic response, with only 1 and 2 isolates (2.8 and 5.6%), respectively. Depending on the sampling time, 36.4% of the isolates from Start Season 1, 54.9% of the isolates from End Season 1, and 22.2% of the isolates from Start Season 2 exhibited antagonism against target fungi. All isolates were identified through PCR (16S rRNA): *Streptomyces* spp. (26), *Bacillus* spp. (4), *Brevundimonas* spp. (2), *Pseudochrobactrum* spp. (2), *Stenotrophomonas* sp. (1), *Alcaligenes* sp. (1).

**Table 2:** Antagonistic activity of soil-isolated bacteria at different sampling times against phytopathogenic and beneficial fungi, and their identification through polymerase chain reaction (PCR) sequencing of amplicons of the 16S rRNA gene.

	Inhibition zone (mm)											
	Pathogenic fungi					Beneficial fungi						
Strain	Forc	Fp	Pc	Botry	Cg	Tl	Th	Ta1	Ta2	Paec	Soil sampling time origin	Identification
20/02-1								6.2 ± 1.6			Start S1	*Bacillus* sp.
20/02-4				5.4 ± 0.5				11.8 ± 1.3			Start S1	*Streptomyces* sp.
20/02-11								4.6 ± 1.1			Start S1	*Alcaligenes* sp.
20/02-22								4.8 ± 1.8			Start S1	*Stenotrophomonas* sp.
20/02-31								4.2 ± 1.6			End S1	*Bacillus* sp.
20/02-33				2.8 ± 0.8	3.0 ± 0.7			7.0 ± 1.2	6.2 ± 2.3		End S1	*Streptomyces* sp.
20/02-35								4.4 ± 1.1			End S1	*Streptomyces* sp.
20/02-39					6.8 ± 1.9			4.0 ± 1.4			End S1	*Streptomyces* sp.
20/02-41	4.6 ± 0.5	3.6 ± 0.9			5.4 ± 1.5						End S1	*Streptomyces* sp.
20/02-48	5.6 ± 0.9	4.0 ± 0.7			9.4 ± 2.3			3.2 ± 0.8		5.0 ± 1.9	End S1	*Streptomyces* sp.
20/02-54				5.0 ± 2.0	3.2 ± 0.8						End S1	*Streptomyces* sp.
20/07-2				8.6 ± 1.9							End S1	*Streptomyces* sp.
20/07-3					6.2 ± 0.8						End S1	*Streptomyces* sp.
20/07-4					2.6 ± 0.9			4.2 ± 0.8			End S1	*Streptomyces* sp.
20/07-9					4.4 ± 0.5						End S1	*Streptomyces* sp.
20/07-13				6.4 ± 0.9	12.8 ± 1.6						End S1	*Streptomyces* sp.
20/07-14				12.6 ± 0.9	8.2 ± 2.0						End S1	*Streptomyces* sp.
A47			6.3 ± 1.2	17.0 ± 1.2			10.4 ± 1.8		8.8 ± 1.1		End S1	*Streptomyces* sp.
A49				19.0 ± 2.5	5.0 ± 2.2		11.8 ± 1.9	7.6 ± 1.7	9.4 ± 0.9		End S1	*Streptomyces* sp.
A51				17.8 ± 0.8	7.6 ± 1.3				4.4 ± 0.5		End S1	*Pseudochrobactrum* sp.
A53				7.8 ± 1.9	6.6 ± 0.5				4.8 ± 0.4		End S1	*Streptomyces* sp.
A54		5.8 ± 2.4									End S1	*Streptomyces* sp.
A56			5.5 ± 1.3		11.0 ± 0.7		12.8 ± 1.3		11.0 ± 1.2		End S1	*Streptomyces* sp.
A57			4.5 ± 3.1		10.6 ± 0.5						End S1	*Brevundimonas* sp.
A60			7.6 ± 1.7		9.6 ± 0.9		12.4 ± 1.1		9.2 ± 0.8		End S1	*Brevundimonas* sp.
A61					4.8 ± 1.3						End S1	*Streptomyces* sp.
A62				6.8 ± 1.0	11.6 ± 1.5			4.4 ± 1.5			End S1	*Streptomyces* sp.
A63			4.0 ± 3.2		4.8 ± 1.8				6.2 ± 2.7		End S1	*Streptomyces* sp.
A83				5.8 ± 0.5	7.8 ± 1.0						Start S2	*Streptomyces* sp.
A88					8.4 ± 1.5						Start S2	*Streptomyces* sp.
A89				4.0 ± 1.7	8.6 ± 1.5						Start S2	*Streptomyces* sp.
A93				7.3 ± 1.5							Start S2	*Streptomyces* sp.
A94			9.0 ± 0.7								Start S2	*Bacillus* sp.
A95					8.4 ± 1.5						Start S2	*Streptomyces* sp.
A99				11.8 ± 1.3							Start S2	*Pseudochrobactrum* sp.
A100	9.8 ± 1.9	10.8 ± 1.5		18.0 ± 2.2	11.8 ± 1.9	13.4 ± 0.9		15.4 ± 0.5	15.8 ± 0.8	8.1 ± 3.0	Start S2	*Bacillus* sp.

Antagonistic activity was considered when the inhibition zone was ≥ 4 mm to any of the target fungus.

Cg: *Colletotrichum gloeosporioides*; Fp: *Fusarium proliferatum*; Forc: *Fusarium oxysporum f.* sp. *radicis-cucumerinum;* Bc: *Botrytis cinerea*; Pc: *Phytophthora capsici*; Th: *Trichoderma harzianum T-22*; Tl: *Trichoderma longibrachiatum*; Ta1: *Trichoderma asperellum*; Ta2: *Trichoderma atroviride*; Paec: *Paecilomyces lilacinus*; S1: season 1, 2019/2020; S2: season 2, 2020/2021.

## 4 Discussion

In this study, the total culturable bacterial populations and thermophilic culturable bacteria experienced shifts depending on the sampling time, but no clear trend was observed and the impact on these bacterial fractions population cannot be conclusively attributed to the treatments. In this regard, it’s important to consider that changes in soil quality parameters and sampling time are factors that highly influence microbial variations, particularly in cultivated and amended soils (Li et al., 2018; Vido et al., 2024).

A noticeable outcome of the study is the dynamics of the bacterial communitys alpha diversity throughout the crop season. Primarily, species richness (Margalef index), but also Shannon diversity and Pielou’s evenness indices, appeared lower at pre-planting phase (Start Season), meanwhile at the end of the cropping seasons, all alpha diversity indices tended to increase and consistently reach higher values. In addition, significant changes in bacterial community beta diversity were recorded across the different sampling times (PERMANOVA *p* < 0.01). In this sense, changes in the soil microbial community have been previously reported after the application of manure in crop fields (Hartmann et al., 2015; Sun et al., 2015; Tang et al., 2023), as well as after solarisation treatments when manure and other organic amendments had been previously incorporated (Achmon et al., 2020; Marín-Guirao et al., 2019b, 2023a; Shea et al., 2022). According to Vido et al. (2024) our results also highlight the dynamic nature of bacterial community composition over time at phylum and genus level when soils are amended with organic materials and organic crop management. In any case, Proteobacteria and Firmicutes were the most prevalent phyla in all sampling times. These two phyla, along with Actinobacteriota, are consistently associated with soil-borne disease suppression (Raaijmakers et al., 2009; Mendes et al., 2011).

In our study, the relative abundance of Firmicutes was higher at pre-planting phase (Start Season) compared to End Season, contrary to Proteobacteria, which consistently showed higher levels at the End Season in both years. Thus, *Bacillus* (phylum Firmicutes) was identified as a biomarker in Start Season, while *Thauera* (phylum Proteobacteria) was the biomarker at the genus level in End Season. These are biomarkers indicating a superior ability to discriminate between the studied conditions and a higher statistical significance and relevance for the study (LDA score >4.5). *Bacillus* is a widely studied genus for its suppressive properties against soil pathogens (Miljaković et al., 2020; Zhang et al., 2023). However, bacteria of the genus *Thauera* have been biotechnologically interesting for their ability to degrade aromatic compounds (Elder and Kelly, 1994; Philipp and Schink, 2000), and more recently for their role in the production of a promising alternative to non-degradable plastics (Koller et al., 2017). To the best of our knowledge, *Thauera* has not been reported to have capabilities for controlling soil pathogens. The observed changes in the abundances of these two genera may have been influenced by the high temperatures reached during the solarization process that followed the incorporation of the organic amendment (Pullman et al., 1981). While, *Bacillus* spp. are recognized thermophilic bacteria (Edwards et al., 1965; Bond and Favero, 1975), most *Thauera* species are recognized as mesophilic bacteria (Fida et al., 2016), although a thermophilic *Thauera* species has been also reported (Yang et al., 2018). In fact, the treatments did not completely eliminate *Thauera* bacteria but maintained a viable inoculum capable of recolonizing the soil during the crop season. In this regard, the incorporation of an organic amendment rich in nitrogen, such as fresh sheep manure, could favor the presence of these bacteria in the studied soil. Both bacteria genera are active microorganisms involved in the nitrogen cycle and have been recognized as dominant denitrifiers in various environments, including soils (Fuka et al., 2007). In this regard, we identified ecological (functional) pathways related to the nitrogen cycle (nitrate and nitrite respiration, nitrate reduction, and ureolysis) that present a higher percentage of functional related sequences at the End of the Season compared to the Start of the Season, immediately after the treatments. As previously reported, alkeline hydrolyzable organic nitrogen content and soil organic carbon content are key factors that drive shifts in soil microbial composition and could also determine the observed changes in microbial functional communities formed at different successional stages (Dasgupta et al., 2024; Ding et al., 2024).

A relevant result of the present study is the presence of native bacteria with antagonistic properties against prevalent pathogenic fungi, but also against common beneficial fungi sourced from commercial products. Even a large number of these isolates exhibited antagonism against both pathogenic and beneficial fungi, thus highlighting the complexity of the edaphic system, an aspect often overlooked in agricultural soil management plans. In addition, isolates with antagonistic activity were detected at all sampling times throughout the study, both following the treatments performed and at the end of the crop season, which suggests that they are also present during the development of the crops. Among the native antagonistic bacteria (16S rRNA), *Streptomyces* was the dominant genus followed by *Bacillus*, while bacteria from the genera *Brevundimonas*, *Pseudochrobactrum*, *Stenotrophomonas*, and *Alcaligenes* also showed antagonism. Among them, *Streptomyces* spp. and *Bacillus* spp. are among the most studied biological control agents. Their role in the suppression of several plant pathogens has been well-documented and attributed to their production of a wide variety of bioactive secondary metabolites and enzymes, including antibiotics, bacteriocins, and volatile compounds (Alabouvette and Steinberg, 2006; Kunova et al., 2016; Zhang et al., 2023).

The evaluation of the specific soil management practices needed for developing and maintaining soil microbial functionality which favor DSS in cultivable lands is a highly complex issue that is difficult to decipher (Alabouvette and Steinberg, 2006). It must be considered that the complexity of the microbial interactions as well as the underlying mechanisms and microbial traits remain elusive for most disease suppressive soils (Gómez Expósito et al., 2017). In any case, in our study, isolates with antagonistic activity were detected at all sampling times, even after incorporating fresh sheep manure in summer followed by solarisation for 2 months. All the isolates used in the antagonism assays, were obtained from the thermophilic bacteria analyses performed in this study, and thus their presence is high in the evaluated soil, with populations ranging from millions to tens of millions of CFU/g dry soil. Thus, the soil management described in this study was compatible with, and possibly responsible for the high presence of these microorganisms in the soil of the greenhouse. This is a highly relevant information for the development of a soil suppressive to soil pathogens, but also for the use of certain commercial products containing BCO and other beneficial microorganisms for crops, as the presence of native bacteria with these properties can influence their effectiveness.

In conclusion, the results of this study show changes in the soil bacterial community and its functionality over the crop cycle following the application of sheep manure combined with summer solarisation in a Mediterranean greenhouse. The study reflects the presence of native bacteria with antagonistic properties inhabiting the mentioned biosolarised soils. Moreover, this fact also demonstrates the differential antagonistic ability of each isolate concerning the phytopathogenic or beneficial profile of the tested fungal species. These results are highly relevant for the application of agronomic soil management practices aimed at improving the natural suppressiveness of soils, to diseases caused by fungi and actinomycetes, as well as for the proper use of formulations based on beneficial fungi. However, further studies are necessary to elucidate whether the suppressive properties against soil-borne pathogens of the studied native bacteria are clearly manifested when considering the intricate interactions among soil, plant, microbiome, and environment. Additionally, these results should guide the development of commercial formulations with microorganisms that ensure farmers the establishment of these microorganisms in their soil and the derived benefits.

## Data Availability

The datasets and sequences from this study are available in the Zenodo open-access repository under the OPTIMSOIL project (PID2021-125545OR-C21): https://zenodo.org/records/15545241.
