# Supplementary material for: Native bacteria from a Mediterranean greenhouse associated to soil health and suppressiveness
Source: Front Microbiol. 2025 Jun 27;16:1484219. doi: 10.3389/fmicb.2025.1484219 (PMC12246977; doi:10.3389/fmicb.2025.1484219)
Supplement: Supplementary file 1 [file Table_1.docx]

**Table S1.** Global PERMANOVA test and PERMANOVA pairwise tests values for the effect of sampling time on bacterial communities.

| **Global test** | **Pseudo-F** | ***P*-value** | **Unique permutations** |
| --- | --- | --- | --- |
| Sampling time | 9.6531 | 0.0001 | 9878 |
| **Pairwise tests** | **t-statistic** | ***P*-value** | **Unique permutations** |
| Start Season 1 vs Start Season 2 | 2.106 | 0.0019 | 455 |
| Start Season 1 vs End Season 1 | 2.1736 | 0.0024 | 455 |
| Start Season 1 vs End Season 2 | 2.3451 | 0.0025 | 455 |
| Start Season 2 vs End Season 1 | 3.7583 | 0.0001 | 9879 |
| Start Season 2 vs End Season 2 | 2.9719 | 0.0001 | 9861 |
| End Season 1, vs End Season 2 | 3.7334 | 0.0001 | 9875 |

**Table S2.** Global PERMANOVA test and PERMANOVA pairwise tests values for the effect of sampling time on the composition and diversity of the top 15 bacterial ecological groups.

| **Global test** | **Pseudo-F** | ***P*-value** | **Unique permutations** |
| --- | --- | --- | --- |
| Sampling time | 31.467 | 0.0001 | 9929 |
| **Pairwise tests** | **t-statistic** | ***P*-value** | **Unique permutations** |
| Start Season 1 vs Start Season 2 | 1,3073 | 0,1096 | 455 |
| Start Season 1 vs End Season 1 | 5,1273 | 0,0025 | 455 |
| Start Season 1 vs End Season 2 | 3,6159 | 0,0022 | 455 |
| Start Season 2 vs End Season 1 | 8,5011 | 0,0001 | 9881 |
| Start Season 2 vs End Season 2 | 6,2318 | 0,0001 | 9893 |
| End Season 1, vs End Season 2 | 3,6677 | 0,0008 | 9905 |
